# Laryngeal tuberculosis presenting as a supraglottic carcinoma: a case report and review of the literature

**DOI:** 10.1186/1752-1947-3-9288

**Published:** 2009-11-20

**Authors:** Yvette E Smulders, Bert-Jan De Bondt, Martin Lacko, Janice AL Hodge, Kenneth W Kross

**Affiliations:** 1 Department of Otolaryngology/Head and Neck Surgery, Maastricht University Medical Centre, the Netherlands; 2 Department of Radiology, Isala Klinieken, Zwolle, the Netherlands; 3 Department of Pathology, Maastricht University Medical Centre, the Netherlands

## Abstract

**Introduction:**

Laryngeal tuberculosis used to be a common complication in advanced pulmonary tuberculosis. However, it has become a rare occurrence in developed countries since the introduction of antituberculous agents. Moreover, the pattern of the disease has changed over the years. Nowadays, it more closely resembles a laryngeal carcinoma than any other laryngeal illness.

**Case presentation:**

We describe the case of a 50-year-old Caucasian man who presented with the clinical picture of laryngeal cancer, but which turned out to be tuberculosis. We illustrate the difficulty of recognizing laryngeal tuberculosis both clinically and even with radiological examination.

**Conclusion:**

Although laryngeal tuberculosis is uncommon, especially in developed countries, it still occurs and should be considered as a differential diagnosis in any laryngeal disease, in particular in the case of a laryngeal carcinoma.

## Introduction

Tuberculosis (TB) was a highly prevalent disease in humans for many decades until the early 1900s and is still the main cause of death in some parts of the world. In 2007, approximately 13.7 million people contracted TB and 1.8 million died as a result of the disease. A total of 68% of newly infected people were of Asian or African origin, whereas ethnic Europeans only made up 5% of global TB cases [1].

**Figure 1:**
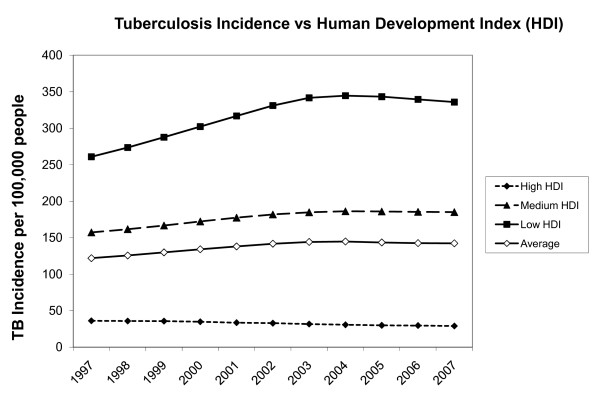
**TB incidence per 100,000 inhabitants for developed, developing and underdeveloped countries in the past decade**. Based on the Human Development Index (HDI) and tuberculosis (TB) incidence per country between 1997 and 2007 [1]. The graph summarizes data of 172 countries: 73 developed countries (HDI >0.8), 73 developing countries (HDI 0.5-0.8) and 26 underdeveloped countries (HDI <0.5).

After the introduction of antituberculous agents, preventive programs and better socioeconomic conditions, TB incidence decreased dramatically up until the 1980s [1-4]. In subsequent years, however, the epidemic spread of HIV, illicit drug use and the emergence of multi-drug-resistant mycobacteria have resulted in a resurgence of TB. In 1993, it became the leading cause of death from a single infectious agent. Increased numbers of migration and travelling to and from less-developed countries also contributed to the worldwide spread of TB [3-7]. The incidence of TB continues to increase (122 per 100,000 in 1997 versus 142 per 100,000 in 2007), despite the fact that it has stabilized or decreased in over 70% of countries. A minority of underdeveloped, mainly African, countries has been the major contributor to the global incidence growth [1]. The change in TB incidence over time for underdeveloped, developing and developed countries is shown in Figure 1.

Laryngeal TB used to be a common complication of pulmonary TB. At the start of the 20th century it affected 25-30% of all infected patients. Today, laryngeal TB occurs in only 1% of cases [4,8-10]. The pattern and clinical symptoms of laryngeal TB have also changed. Currently, it has many similarities to laryngeal carcinoma [4,5,7,11].

We present a case of laryngeal TB in a Caucasian man who had characteristic symptoms of a supraglottic laryngeal carcinoma without any clinical pulmonary manifestations. The purpose of this case report and review of the literature is to raise awareness of this rare but prevailing disease.

## Case presentation

**Figure 2:**
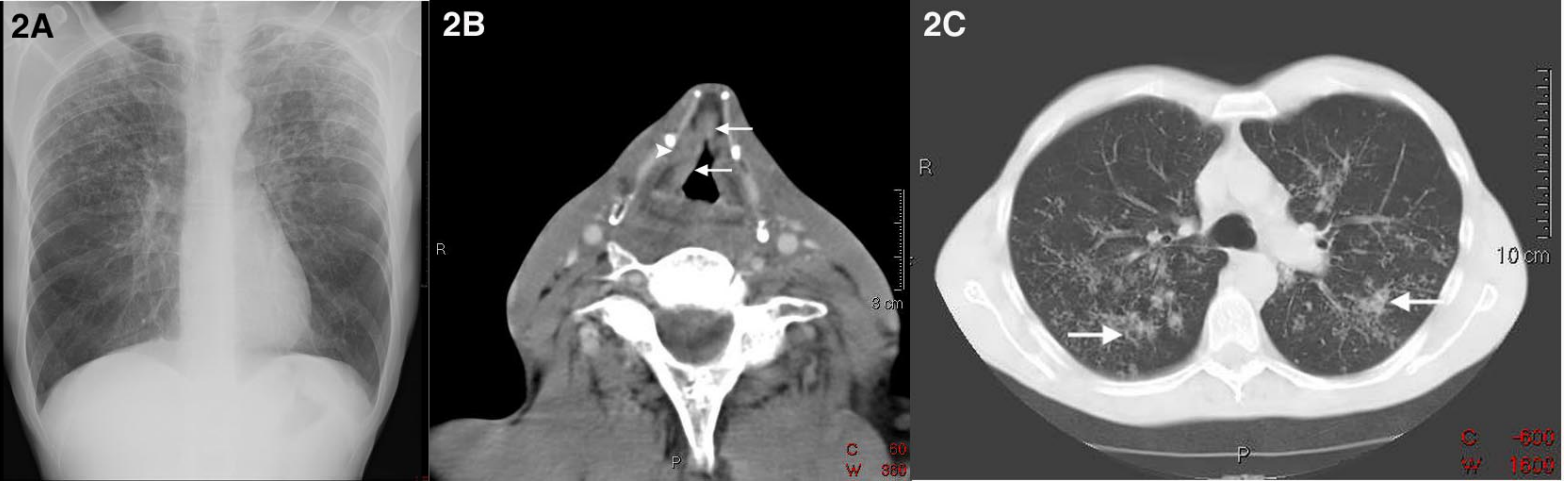
**Radiography**. **(A) **Initial antero-posterior chest X-ray. Miliary nodules predominantly bilaterally distributed in the upper parts of the lung parenchyma. **(B) **Axial contrast-enhanced computed tomography image of the neck: enhanced mass (arrows) in the false vocal cord which extends into the anterior commissure and obliteration of the paraglottic fat (arrowhead). **(C) **Axial high resolution computed tomography image of the chest: diffusely distributed interstitial nodular alterations with formation of central cavities (arrows).

A 50-year-old Caucasian man was referred to our otolaryngology outpatient clinic with a two-month history of persistent dysphonia, dysphagia and odynophagia with radiating pain to the right ear. In addition, he had experienced recurrent aspiration of liquids and had lost 3 kg in weight. His prior medical history was unremarkable. He had been a tobacco smoker for 40 years, but there was no history of alcohol abuse. Laryngoscopy revealed an ulcer on the right false vocal cord with an extension to the epiglottis. Oedema and decreased mobility of the right true vocal cord were observed as well as the diffused swelling of both arytenoids. There was no lymphadenopathy as confirmed by an ultrasound sonography of the neck. The initial anteroposterior chest X-ray, being part of the routine diagnostic work-up, showed miliary nodules that were predominantly bilaterally distributed in the upper parts of the lung parenchyma (Figure 2A). The axial contrast-enhanced computed tomography (CT) scan of the neck demonstrated an enhanced mass in the right false vocal cord with an obliteration of the paraglottic fat, extending into the anterior commissure and cranially, obliterating the right piriform sinus (Figure 2B). A full blood count and electrolytes were within normal limits. Direct laryngoscopy performed under general anaesthesia revealed a tumour covering the entire right, false vocal cord with an extension to the laryngeal side of the epiglottis without affecting the glottis. The tumour was clinically staged as a T3N0 supraglottic laryngeal carcinoma.

**Figure 3:**
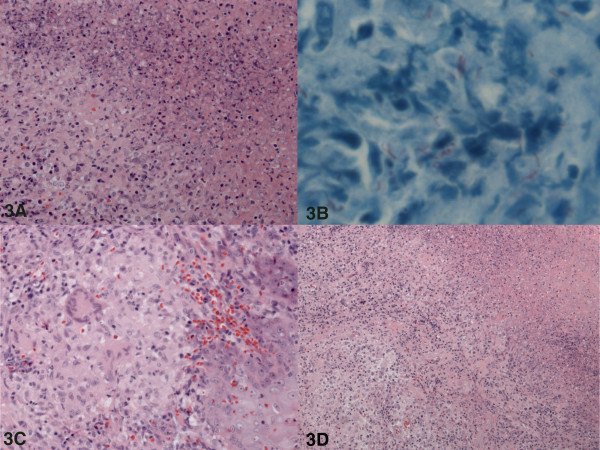
**Biopsy histology**. **(A) **Necrotising granulomatous inflammation: granuloma on the left and necrosis on the right side of the image. **(B) **Red stained *Mycobacterium tuberculosis *in Ziehl-Neelsen acid fast stain. **(C) **Granuloma with a multinucleated giant cell in the center (left side of image). **(D) **Overview of a necrotising granulomatous inflammation: granulomas on the left side and necrosis on the right.

Histopathological examination, however, revealed a necrotising granulomatous inflammation without signs of malignancy (Figure 3A, C, D). Ziehl-Neelsen staining of the tissue biopsies and sputum revealed acid-fast bacilli (Figure 3B). Sputum culture turned out to be positive for *Mycobacterium tuberculosis*. An additional axial high resolution CT scan of the chest demonstrated diffuse interstitial nodular alterations with the formation of central cavities, indicative of pulmonary TB (Figure 2C).

Treatment commenced with isoniazid 300 mg/day, rifampicin 600 mg/day, pyrizinamide 1500 mg/day and ethambutol 1400 mg/day for a period of 2 months. Sputum cultures became negative after a 3-month follow up. Because of cavity formations, as revealed by the chest CT, treatment with isoniazid and rifampicin was prolonged for another 3 months.

Because the patient was discharged from hospital a day after a direct laryngoscopy, there was ample opportunity for the patient to infect his healthcare providers. Contact surveys revealed that the resident and nurse, who both assisted in performing the laryngoscopy, two of the patient's relatives and two of his friends all tested positive in their purified protein derivative tests (PPD). They were all preventively treated with tuberculostatics.

## Discussion

Extra pulmonary TB in the head and neck region most frequently occurs in the cervical lymph nodes (>90%), followed by the larynx (2% to 6%) [8,9]. Involvement of the temporal bone, sinonasal cavity, eye, pharynx, thyroid and skull base are even less frequently observed [4,8,9]. The characteristics of laryngeal TB have changed over the years and it has become a challenge for otolaryngologists to distinguish this disease from others. In the past, laryngeal TB typically affected young people in the second or third decade of life with advanced pulmonary TB. Symptoms were cough, haemoptysis, fever, weight loss and night sweats. An ulcerative, granulomatous lesion was generally positioned on the posterior part of the larynx due to accumulation of sputum in the arytenoid region in bed-bound patients. Today, laryngeal TB mainly involves people in their 50 s or 60 s presenting first and foremost with hoarseness (80% to 100%). Other symptoms are odynophagia (50% to 67%) and to a lesser extent, dysphagia, dyspnoea, stridor, cough and haemoptysis.

Systemic symptoms have become rare [2,4-7,9,11]. Laryngeal TB can involve all parts of the larynx and there is no longer an unmistakable association with pulmonary TB. The larynx becomes infected either by a direct spread from the lungs, or by a haematogenous spread from sites other than the lungs [5,11,12]. The former mechanism is most common and probably relevant for the patient in our case. In the case of a haematogenous spread, there is no evidence of pulmonary disease [3,7,9]. The distinction between laryngeal TB and chronic laryngitis or laryngeal carcinoma in particular has become difficult. Odynophagia is described as an important discriminating symptom, since it is considered rare in laryngeal cancer [2,5-7,11]. Yet, from experience we know that painful dysphagia is a well-known symptom reported among patients suffering from a supraglottic laryngeal carcinoma. In a physical examination, the true vocal cords are most frequently affected by laryngeal TB, followed by the epiglottis, false vocal cords and ventricles, arytenoids, posterior commissure and the subglottic area [4,7,12]. Laryngeal TB can manifest as oedema, hyperaemia or ulcerative lesions, but can also present as a nodule, an exophytic mass or obliteration of an anatomical structure [12]. Aside from chronic laryngitis and laryngeal carcinoma, these various presentations give rise to a comprehensive differential diagnosis including cat-scratch disease, syphilis, sarcoidosis, Wegener's granulomatosis and fungal infections [8]. Since pathognomonic characteristics indicative of laryngeal TB do not exist and the fact that it is an uncommon disease in industrialised countries, the infection is easily mistaken for the more frequently occurring laryngeal carcinoma [3,6,8]. Our patient had a long-standing history of heavy smoking, and had symptoms of hoarseness and odynophagia. Laryngoscopy revealed an oedematous tumour with decreased mobility of the vocal cord. The clinical signs, supported by the findings on the initial CT scan of the larynx, led us to the diagnosis of a laryngeal carcinoma. Laryngeal TB, however, can have the exact same symptoms. In the majority of cases, there is an association with pulmonary TB [2,4]. Therefore, an anomalous chest X-ray, if not compatible with pulmonary metastasis, should alert the radiologist and otolaryngologist to the possibility of TB, especially when former chest X-rays were normal.

Laboratory techniques for detecting TB infections include histopathological tissue examinations with Ziehl-Neelsen histochemical staining for acid-fast bacilli and identification of *M. tuberculosis *by polymerase chain reaction or bacterial culture. The latter method, although time-consuming, is considered the reference standard [13]. A CT of the neck cannot definitively identify laryngeal TB since, as in a chest X-ray, it can imitate many other diseases. Antituberculous agents are the primary treatment for laryngeal TB. The improvement of dysphagia and resolving of cavernous lung lesions are expected to occur within several weeks [6,12,14,15]. If not treated early, laryngeal TB can result in (sub)glottic stenosis, muscular involvement and vocal cord paralysis when the cricoarytenoid joint or recurrent laryngeal nerve are invaded [5,12].

## Conclusion

Laryngeal TB is uncommon, particularly in developed countries, but it still occurs. There are no pathognomonic features indicative of this disease and it can mimic many others. If misdiagnosed, laryngeal TB can have severe consequences for the patient and anyone he comes into contact with. Therefore, it is important for otolaryngologists to recognise the altered pattern of laryngeal TB and to be familiar with its resemblance to malignancy. This is not only in view of clinical symptoms, but also from a radiological point of view. Laryngeal TB should be considered as a differential diagnosis in any laryngeal disease and in particular in the case of a laryngeal carcinoma.

## Abbreviations

CT: computed tomography; PPD: purified protein derivative tests; TB: tuberculosis.

## Competing interests

The authors declare that they have no competing interests.

## Authors' contributions

KK, YS and ML were in charge of the medical care of the patient. RB was responsible for the interpretation of the radiological findings and JH for the interpretation of the histopathological findings. They cooperated with each other to find the correct diagnosis and treatment. YS wrote the manuscript and studied the literature. KK, JH and RB reviewed the draft critically and provided helpful comments. All authors read and approved the final manuscript.

## Consent

Written informed consent was obtained from the patient for publication of this case report and any accompanying images. A copy of the written consent is available for review by the Editor-in-Chief of this journal.
